# Strongly Bound Polynuclear Anions Comprising Scandium Fluoride Building Blocks

**DOI:** 10.1021/acs.inorgchem.3c02937

**Published:** 2023-10-02

**Authors:** Iwona Anusiewicz, Piotr Skurski

**Affiliations:** † Laboratory of Quantum Chemistry, Faculty of Chemistry, University of Gdańsk, Wita Stwosza 63, 80-308 Gdańsk, Poland; ‡ Department of Chemistry, University of Utah, Salt Lake City, Utah 84112, United States; § QSAR Lab Ltd., Trzy Lipy 3, 80-172 Gdańsk, Poland

## Abstract

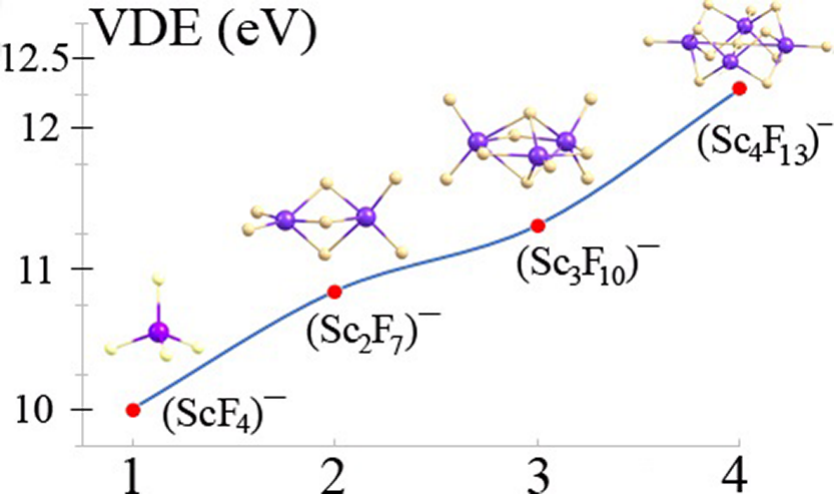

The stability of polynuclear anions composed of ScF_3_ building blocks was studied by using ab initio and density functional theory electronic structure methods and flexible basis sets. Thorough exploration of ground state potential energy surfaces of (Sc_2_F_7_)^−^, (Sc_3_F_10_)^−^, and (Sc_4_F_13_)^−^ anions which may be viewed as comprising ScF_3_ fragments and the additional fluorine atom led to determining the isomeric structures thereof. It was found that the most stable isomers which are predicted to dominate at room temperature correspond to the compact structures enabling the formation of a large number of Sc–F–Sc bridging linkages rather than to the chain-like structures. The vertical electron detachment energies of the (Sc_*n*_F_3*n*+1_)^−^ anions were found to be very large (spanning the 10.85–12.29 eV range) and increasing with the increasing number of scandium atoms (*n*) and thus the ScF_3_ building blocks involved in the structure. Thermodynamic stability of (Sc_*n*_F_3*n*+1_)^−^ anions (i.e., their susceptibility to fragmentation) was also verified and discussed.

## Abstract 2

The anions matching the (Sc_*n*_F_3*n*+1_)^−^ formula are thermodynamically stable systems, not susceptible to fragmentations yielding either ScF_3_ or F^−^ products. The vertical electron detachment energies of the (Sc_*n*_F_3*n*+1_)^−^ (*n* = 1−4) anions span the 10.01−12.29 eV range and increase when *n* develops from 1 to 4.

## Introduction

There is considerable interest in studying superhalogen anions not only because they represent molecular systems exhibiting very large excess electron binding energies but also they play an important role in ionic liquids by acting as their negatively charged building blocks.^1,2^ In addition, superhalogens are being employed in the preparation of many new materials, including organic metals and organic superconductors, hydrogen storage materials, solar cells, and Li-ion batteries.^3−7^ The term “superhalogen” was first used in 1981 by Gutsev and Boldyrev who confirmed the stabilities and large excess electron binding energies of several systems matching the (MX_*k*+1_)^−^ formula (where M is a central metal atom of maximal valence *k*, whereas X stands for halogen atom).^8^ As recognized during many other studies that followed, the (MX_*k*+1_)^−^ formula is much more general than it originally appeared. Namely, it was demonstrated that various molecular fragments (such as acidic functional groups,^9^ halogenoids,^10^ electrophilic groups,^11^ and even superhalogens themselves^12−15^) may act as suitable ligands X, whereas certain nonmetal atoms (e.g., phosphorus,^16^ silicon,^17−20^ hydrogen^21,22^) can play the central atom M role (see the recent review article^23^).

The extension of the superhalogen formula led to defining the polynuclear (M_*n*_X_*n·k+*1_)^−^ superhalogen anions (e.g., (Ca_2_(CN)_5_)^−^, (Na_4_Cl_5_)^−^), whose various applications are constantly being discovered.^24−38^ However, theoretical studies aimed to determine the properties of such species are difficult due to the large number of isomeric structures that must be considered and characterized. Albeit several (M_*n*_X_*n·k+*1_)^−^ negatively charged systems have already been described in the literature,^23^ many more polynuclear superhalogen anions are still waiting to be discovered. In particular, reports describing the (M_*n*_X_*n·k+*1_)^−^ anions containing transition and rare-earth-metal atoms are very scarce.

In this contribution, we present the results of our theoretical investigation concerning polynuclear superhalogen anions containing scandium (historically classified as a rare-earth element) decorated with fluorine ligands. Since scandium chemistry is almost completely dominated by the compounds involving Sc^3+^ trivalent cation (with scandium oxide (Sc_2_O_3_) and scandium fluoride (ScF_3_) as prominent examples),^39−41^ it is commonly assumed that the scandium most predominant oxidation state is +3. Indeed, as demonstrated by Pradhan et al., (ScX_4_)^−^ tetrahedral anions exhibit the largest vertical electron detachment energies (VDEs) among the (ScX_*n*_)^−^ (X = F, Cl, Br; *n* = 1–5) systems considered.^42^ Therefore, we decided to investigate the (Sc_*n*_F_3*n*+1_)^−^ anions (for *n* = 2–4) which can be viewed as comprising *n* ScF_3_ moieties and one additional fluorine atom. To the best of our knowledge, the results concerning the (Sc_2_F_7_)^−^, (Sc_3_F_10_)^−^, and (Sc_4_F_13_)^−^ anions have not been reported in the literature thus far. We believe that providing the knowledge of the most stable isomeric structures of (Sc_*n*_F_3*n*+1_)^−^ anions (*n* = 2–4) and their excess electron binding energies will assist the researchers in designing new scandium-containing alloys and other scandium-doped materials.

## Methods

### Preselection of the Isomeric Structures

The search for the low-energy isomeric structures of (Sc_*n*_F_3*n*+1_)^−^ anions (*n* = 2–4) and their corresponding neutral Sc_*n*_F_3*n*+1_ parents was carried out (for each *n*) using the Coalescence Kick (CK) method.^43,44^ In the CK procedure, a large number of random structures are initially generated. Since these initial random structures often consist of nonbonded molecular fragments, the fragmented parts are simultaneously brought together (by pushing them to the center of mass) to achieve a so-called “coalescence” (connectivity). Once each obtained structure is checked for connectivity, the preliminary geometry optimization process begins. In our case, the CK procedure assumed the initial optimizations with the B3LYP method^45,46^ together with the Los Alamos National Laboratory (LANL) effective core potentials (ECPs) with the appropriate valence basis set of double-ζ quality (denoted LANL2DZ).^47−49^

For each (Sc_*n*_F_3*n*+1_)^−^ and Sc_*n*_F_3*n*+1_ (*n* = 2–4) system considered, ca. 2000–3000 initial structures were generated, coalesced with the CK, and then transformed to represent the nearest local minima by performing the geometry optimization at the B3LYP/LANL2DZ level for each trial structure. In such a way, the CK technique allowed us to preselect the lowest energy isomers in all (Sc_*n*_F_3*n*+1_)^−^ and Sc_*n*_F_3*n*+1_ (*n* = 2–4) cases, which were further investigated at a more reliable level of theory.

### Refinement of the Isomeric Structures

The (Sc_*n*_F_3*n*+1_)^−^ and Sc_*n*_F_3*n*+1_ (*n* = 2–4) isomeric structures preselected by using the CK procedure were refined by applying the density functional theory with the ωB97XD long-range-corrected functional including empirical dispersion^50^ together with the aug-cc-pVDZ basis set^51,52^ for fluorine atoms and Stuttgart RSC 1997 effective core potential (also known as Stuttgart/Dresden effective core potential, SDD) for scandium atoms.^53−55^ In each case, the harmonic vibrational frequencies characterizing the stationary point structure were evaluated at the same level of theory to ensure that all obtained structures correspond to true minima on the potential energy surface.

For the selected anionic isomers investigated, namely, three negatively charged (Sc_2_F_7_)^−^ systems and six isomeric (Sc_3_F_10_)^−^ structures, we determined the stationary point structures at both ωB97XD/SDD/aug-cc-pVDZ and MP2/Sapporo-QZP-diffuse theory levels (where MP2 stands for the second-order Mo̷ller-Plesset perturbation method (MP2),^56−58^ whereas Sapporo-QZP-diffuse indicates the all-electron quadrupole-zeta Sapporo basis set supplemented with diffuse functions for Sc^59,60^ and F^61,62^) to verify whether the former theoretical treatment provides the bond lengths and valence and dihedral angles that can be considered reliable. As it turned out, the structures obtained at the ωB97XD/SDD/aug-cc-pVDZ level differ from those determined at the MP2/Sapporo-QZP-diffuse level only slightly (i.e., the latter treatment leads to bond lengths longer by less than 0.11 Å than those predicted by the former approach, whereas the valence angles obtained at the ωB97XD/SDD/aug-cc-pVDZ theory level differ by less than 3° than those predicted by the MP2/Sapporo-QZP-diffuse treatment. Therefore, we are confident that the ωB97XD/SDD/aug-cc-pVDZ theory level we applied to obtain the isomeric structures of (Sc_*n*_F_3*n*+1_)^−^ and Sc_*n*_F_3*n*+1_ (*n* = 2–4) systems is sufficient.

### Refinement of the Electronic Energies

The electronic energies of the (Sc_*n*_F_3*n*+1_)^−^ and Sc_*n*_F_3*n*+1_ (*n* = 2–4) isomeric structures obtained at the ωB97XD/SDD/aug-cc-pVDZ theory level were refined by employing the coupled-cluster method with the single and double excitations (CCSD)^63−65^ using the same SDD/aug-cc-pVDZ basis sets.

In order to verify whether the relative energies of the isomers obtained at the CCSD/SDD/aug-cc-pVDZ theory level can be considered reliable, we performed additional calculations of the relative energies of the (Sc_2_F_7_)^−^ isomers using the same CCSD method with the all-electron Sapporo-QZP-diffuse basis set. As it turned out, the energy order of the isomers remains the same regardless the basis sets employed while the differences in relative energies are larger by ca. 0.09–0.13 eV when the CCSD/Sapporo-QZP-diffuse treatment is applied (in comparison to the relative energies obtained at the CCSD/SDD/aug-cc-pVDZ theory level). In addition, we calculated the relative energies of six isomeric (Sc_3_F_10_)^−^ structures by employing the CCSD/Sapporo-TZP-diffuse treatment to verify whether the corresponding relative energies obtained at the CCSD/SDD/aug-cc-pVDZ theory level can be considered reliable. As it turned out, the relative energies obtained for the (Sc_3_F_10_)^−^ isomers at the CCSD/Sapporo-TZP-diffuse theory level differ from those obtained at the CCSD/SDD/aug-cc-pVDZ theory level by 0.04–0.13 eV. Therefore, we are confident that the refined electronic energies of the (Sc_*n*_F_3*n*+1_)^−^ and Sc_*n*_F_3*n*+1_ isomers (*n* = 2–4) and thus their relative energies we predicted by employing the CCSD/SDD/aug-cc-pVDZ treatment can be considered reliable.

### Vertical Electron Detachment Energies

The VDEs characterizing the isomers of the (Sc_*n*_F_3*n*+1_)^−^ anions (*n* = 2–4) were calculated by applying the outer valence Green function OVGF method (*B* approximation)^66−74^ together with the Stuttgart RSC 1997 effective core potential for scandium and the aug-cc-pVDZ basis set for fluorine. Due to the fact that the OVGF approximation remains valid only for outer valence ionization for which the pole strengths (PS) are greater than 0.80–0.85,^75^ we verified that the PS values obtained were sufficiently large (i.e., spanning the 0.930–0.938 range) to justify the use of the OVGF method.

The adiabatic electron affinities of the Sc_*n*_F_3*n*+1_ neutral systems (*n* = 2–4) were obtained by subtracting the CCSD/SDD/aug-cc-pVDZ electronic energies of the (Sc_*n*_F_3*n*+1_)^−^ anions from those of their corresponding neutral parents (all determined for the lowest energy isomeric structure obtained at the ωB97XD/SDD/aug-cc-pVDZ theory level).

In addition, we verified whether the VDEs obtained at the OVGF/SDD/aug-cc-pVDZ theory level can be considered reliable by calculating the vertical electron detachment energy for the tetrahedral (ScF_4_)^−^ anion using two approaches: (i) the OVGF method and all-electron Sapporo-QZP-diffuse basis set for Sc and F and (ii) the OVGF method and SDD pseudopotentials for Sc and the aug-cc-pVDZ basis set for F. Since we found that the VDE of the *T*_d_-symmetry (ScF_4_)^−^ anion calculated by applying the OVGF/Sapporo-QZP-diffuse treatment (10.040 eV) is larger by only 0.034 eV than that obtained by using the OVGF/SDD/aug-cc-pVDZ approach (10.006 eV), we believe that our vertical electron detachment energies predicted for the (Sc_*n*_F_3*n*+1_)^−^ (*n* = 2–4) isomers at the OVGF/SDD/aug-cc-pVDZ theory level are reliable yet likely underestimated by ca. 0.5%.

### Additional Remarks

The reaction energies and the Gibbs free reaction energies (at *T* = 298.15 K) for the fragmentation processes of the (Sc_*n*_F_3*n*+1_)^−^ (*n* = 2–4) anions were calculated using the CCSD/SDD/aug-cc-pVDZ electronic energies and zero-point energy corrections, thermal corrections, and entropy contributions estimated at the ωB97XD/SDD/aug-cc-pVDZ theory level.

Since the neutral Sc_*n*_F_3*n*+1_ (*n* = 2–4) systems are open-shell molecules, we used methods based on an unrestricted Kohn–Sham or Hartree–Fock starting point. Hence, it was important to make sure that little (if any) artificial spin contamination enters into the final wave functions. We computed the expectation value ⟨*S*^*2*^⟩ for the states studied in this work and found values not exceeding 0.774 for doublet neutral species (at the unrestricted DFT or HF level). Hence, we are confident that spin contamination is not large enough to significantly affect our findings.

All calculations were performed with the GAUSSIAN16 (Rev.C.01) package.^76^

## Results and Discussion

### Isomeric Structures of (Sc_2_F_7_)^−^ Anions

**Figure 1:**
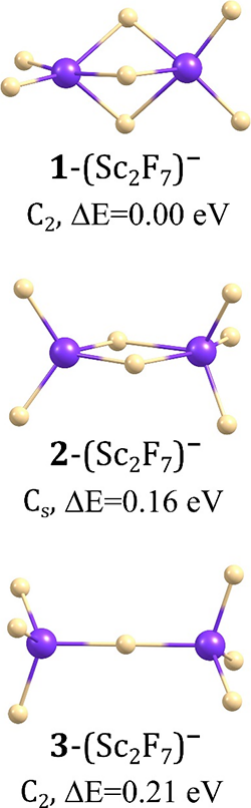
Isomeric structures of the (Sc_2_F_7_)^−^ anion with relative energies (Δ*E*).

The search for the geometrically stable structures of the (Sc_2_F_7_)^−^ anion led to three isomers labeled **1**-**3** in Figure 1. The global minimum (**1**-(Sc_2_F_7_)^−^) corresponds to the *C*_2_-symmetry structure with two ScF_2_ fragments oriented nearly perpendicularly to each other and connected via three F atoms forming a triangle between the scandium atoms. The lengths of the terminal Sc–F bonds (1.883–1.890 Å) are shorter than those of the scandium–fluorine separations in the Sc–F–Sc bridging fragments (1.982–2.245 Å). In the second lowest energy structure (labeled **2**-(Sc_2_F_7_)^−^ in Figure 1), the Sc atoms are connected via two F atoms (with the Sc–F distances spanning the 1.971–2.303 Å range) which leads to the uneven distribution of the remaining ligands between two central atoms. In consequence, the energy of the *C*_s_-symmetry isomer **2**-(Sc_2_F_7_)^−^ is slightly (by 0.16 eV) larger than that of the global minimum. Since the remaining isomer **3**-(Sc_2_F_7_)^−^ whose relative energy (Δ*E*) is even higher (0.21 eV) contains only one Sc–F–Sc connection (see Figure 1) between two ScF_3_ moieties, we conclude that the formation of three Sc–F–Sc bridging fragments is energetically favorable in polynuclear superhalogen anions involving scandium central atoms and fluorine ligands.

### Isomeric Structures of (Sc_3_F_10_)^−^ Anions

In the case of the (Sc_3_F_10_)^−^ anion, we found six isomeric structures whose relative energies do not exceed 1.7 eV. Although we realize that the formation of isomers whose relative energies are large is highly unlikely at room temperature, we decided to include such structures in our discussion to demonstrate that the chain-like isomers in which the fragments are connected via the single Sc–F–Sc bridging linkage are significantly less stable than the structurally compact isomers containing scandium atoms connected through two or three such linkages.

**Figure 2:**
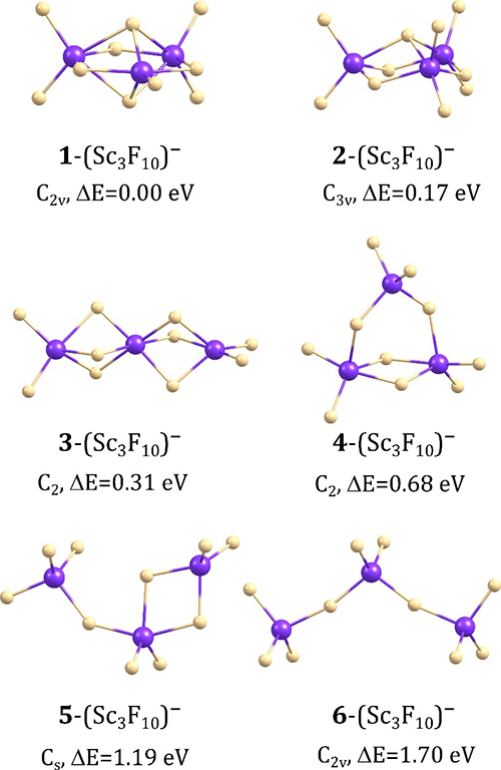
Isomeric structures of the (Sc_3_F_10_)^−^ anion with relative energies (Δ*E*).

The most stable isomer of (Sc_3_F_10_)^−^ anion (labeled **1**-(Sc_3_F_10_)^−^ in Figure 2) corresponds to the compact *C*_2*v*_-symmetry structure containing the cage-like fragment formed by the planar 6-member ring (involving alternately aligned Sc and F atoms connected via the Sc–F bonds whose lengths span the 2.015–2.120 Å range) and two F atoms below and above the ring plane. Such an alignment allows each Sc to be involved in two Sc–F–Sc and two Sc-FSc_2_ motifs, whereas the remaining fluorine ligands are distributed among three Sc atoms and connected to them through shorter (1.868–1.873 Å) bonds. Among the other isomers, the only structure which could be considered competitive (due to its small Δ*E* value of 0.17 eV) with the lowest energy isomer 1 is the *C*_3*v*_-symmetry **2**-(Sc_3_F_10_)^−^ isomer which differs from **1**-(Sc_3_F_10_)^−^ by the position of one fluorine ligand. Namely, instead of being involved in the cage-like fragment as in **1**-(Sc_3_F_10_)^−^, one more F atom is connected to a single Sc atom, thus making six F ligands distributed evenly among three central Sc atoms and sticking out of the 6-member ring. In consequence, the ring itself becomes nonplanar and adopts a chair conformation instead (see Figure 2). The third lowest energy isomer, **3**-(Sc_3_F_10_)^−^, corresponds to a chain-like *C*_2_-symmetry structure with scandium atoms connected via three Sc–F–Sc bridging linkages and thus resembles the global minimum identified for (Sc_2_F_7_)^−^ anion (cf. **1**-(Sc_2_F_7_)^−^ and **3**-(Sc_3_F_10_)^−^, depicted in Figures 1 and 2, respectively). Although the relative energy of **3**-(Sc_3_F_10_)^−^ is larger than that of the global minimum by 0.31 eV, this isomer is substantially more stable than the remaining **4**–**6** isomers of (Sc_3_F_10_)^−^, likely due to the presence of a large number of Sc–F–Sc bridging fragments which stabilize the structure. As far as these remaining higher energy **4**–**6** isomers are concerned, none of them contains a pair of Sc atoms connected via three fluorine ligands. In **4**-(Sc_3_F_10_)^−^ (Δ*E* = 0.68 eV) and **5**-(Sc_3_F_10_)^−^ (Δ*E* = 1.19 eV), only one double Sc–F–Sc linkage can be distinguished, whereas the isomer **6**-(Sc_3_F_10_)^−^ (Δ*E* = 1.70 eV) corresponds to the F_3_Sc–F–ScF_2_–F-ScF_3_ structure containing the scandium atoms connected only through single Sc–F–Sc bridges (nota bene, the structures in which the central atoms are linked via *one* halogen atom were assumed to correspond to the most stable isomers of polynuclear superhalogen anions in early reports concerning these systems^77^).

### Isomeric Structures of (Sc_4_F_13_)^−^ Anions

**Figure 3:**
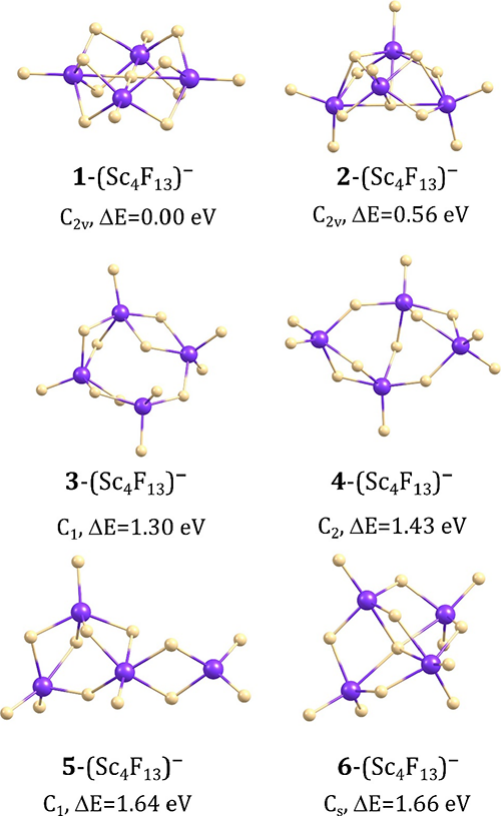
Isomeric structures of the (Sc_4_F_13_)^−^ anion with their relative energies (Δ*E*) (see Figure 4 for the remaining isomers of (Sc_4_F_13_)^−^).

The extensive search of the ground state potential energy surface of the (Sc_4_F_13_)^−^ anion led to three isomeric structures whose relative energies are within 1.3 eV and several isomers whose Δ*E* values span the 1.3–4.1 eV range. As in the preceding section, we decided to briefly mention also these high energy isomers in our discussion because we wanted to include the fully extended chain-like structure while making the comparison of the relative stability. The global minimum (labeled **1**-(Sc_4_F_13_)^−^ in Figure 3) corresponds to the *C*_2*v*_-symmetry compact structure, resembling a double crown with the F atom in its center and four Sc atoms forming a square. Adopting such a structure seems to maximize the number of Sc–F–Sc connections leaving only four fluorine ligands outside that central substructure. The Sc–F bond lengths span the 2.075–2.178 Å range in a double crown fragment, whereas the lengths of the Sc–F bonds sticking out of that substructure are shorter (1.860 Å).

**Figure 4:**
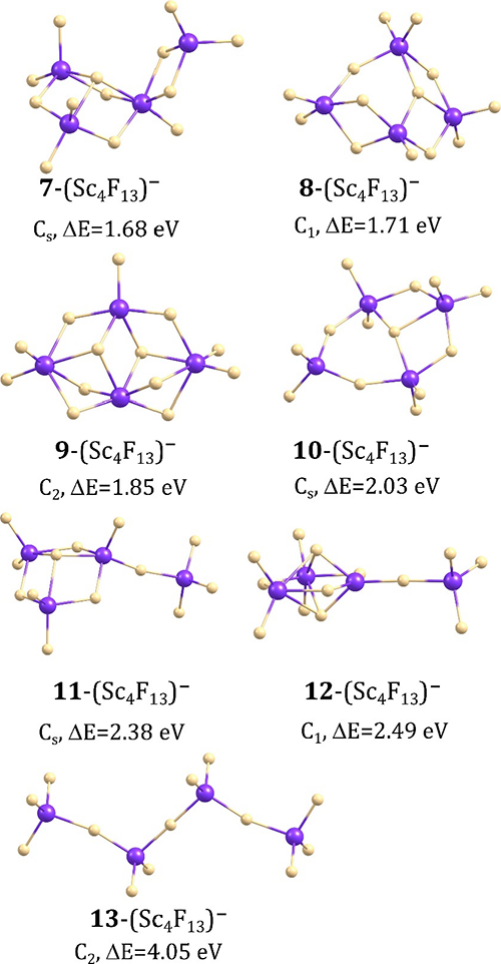
Isomeric structures of the (Sc_4_F_13_)^−^ anion with their relative energies (Δ*E*) (see Figure 3 for the lower energy isomers of that system).

The second lowest energy isomer (**2**-(Sc_4_F_13_)^−^) also corresponds to the *C*_2*v*_-symmetry compact structure yet with a different (i.e., nonplanar) mutual alignment of the Sc atoms. In consequence, the number of Sc–F–Sc bonds in **2**-(Sc_4_F_13_)^−^ is smaller than that in **1**-(Sc_4_F_13_)^−^ which results in the higher electronic energy of the former (Δ*E* = 0.56 eV). Such a large energy gap between the global minimum **1** and the local minimum **2** indicates that the formation of any other isomeric structure of (Sc_4_F_13_)^−^ but **1**-(Sc_4_F_13_)^−^ should be considered highly unlikely at room temperature. Apart from these two low-energy isomers, we found several other geometrically stable structures of (Sc_4_F_13_)^−^ having much larger relative energies in the range of 1.30–2.49 eV (see the isomers labeled **3**–**12** in Figures 3 and 4). Alike in the (Sc_2_F_7_)^−^ and (Sc_3_F_10_)^−^ cases, the fully extended isomer of the (Sc_4_F_13_)^−^ anion (i.e., containing the scandium atoms linked via one fluorine ligand, see **13**-(Sc_4_F_13_)^−^ in Figure 4) has a very large relative energy (4.05 eV).

### Excess Electron Binding Energies of (Sc_*n*_F_3*n*+1_)^−^ Anions (*n* = 1–4)

Since the (Sc_*n*_F_3*n*+1_)^−^ anions (*n* = 2–4) can be viewed as obtained by subsequent attachments of the ScF_3_ fragment to the (ScF_4_)^−^ anion (matching the (Sc_*n*_F_3*n*+1_)^−^ formula for *n* = 1), we include the (ScF_4_)^−^ system in our discussion of the electronic stability. In general, it should be emphasized that the vertical electron detachment energies of all (Sc_*n*_F_3*n*+1_)^−^ anions (*n* = 1–4) are very large, as even the VDE of (ScF_4_)^−^ exceeds 10 eV. Next, the results gathered in Table 1 indicate that the VDEs of the (Sc_*n*_F_3*n*+1_)^−^ anions increase when *n* develops from 1 to 4. Namely, taking into account all isomeric structures, we calculated the VDEs to span the 10.44–11.48 eV range (for *n* = 2), the 11.31–12.06 eV range (for *n* = 3), and the 11.44–12.38 eV range (for *n* = 4). Certainly, the increase in the VDE for (Sc_*n*_F_3*n*+1_)^−^ anions as *n* develops from 1 to 4 can be attributed to the growing number of fluorine ligands. Since we predicted only the lowest energy isomer of each anion to exist at room temperature, it seems reasonable to compare their VDE values as representative for polynuclear (Sc_*n*_F_3*n*+1_)^−^ superhalogen anions of different size. Such a comparison reveals that attaching the first ScF_3_ subunit to (ScF_4_)^−^ increases the VDE by 0.84 eV, whereas the subsequent ScF_3_ fragment attachments further increase the VDE by 0.47 eV and then by 0.97 eV, leading to the **1**-(Sc_2_F_7_)^−^, **1**-(Sc_3_F_10_)^−^, and **1**-(Sc_4_F_13_)^−^ anions having their VDEs of 10.85, 11.32, and 12.29 eV, respectively. Such an increase of the excess electron binding energy with the increasing number of ScF_3_ fragments involved in the structure seems very large and we suspect that even larger vertical electron detachment energies could be achieved for the (Sc_*n*_F_3*n*+1_)^−^ superhalogen anions with *n* > 4.

**Table 1:** Vertical Electron Detachment Energies (in eV) and Relative Energies (Δ*E* in eV) of Isomeric Structures of (Sc_*n*_F_3*n*+1_)^−^ Anions (*n* = 1–4)^a^

system	Δ*E*	VDE
(ScF_4_)^−^	0.00	10.01
**1**-(Sc_2_F_7_)^−^	0.00	10.85
**2**-(Sc_2_F_7_)^−^	0.16	10.44
**3**-(Sc_2_F_7_)^−^	0.21	11.48
**1**-(Sc_3_F_10_)^−^	0.00	11.32
**2**-(Sc_3_F_10_)^−^	0.17	11.80
**3**-(Sc_3_F_10_)^−^	0.31	11.53
**4**-(Sc_3_F_10_)^−^	0.68	11.31
**5**-(Sc_3_F_10_)^−^	1.19	11.45
**6**-(Sc_3_F_10_)^−^	1.70	12.06
**1**-(Sc_4_F_13_)^−^	0.00	12.29
**2**-(Sc_4_F_13_)^−^	0.56	11.80
**3**-(Sc_4_F_13_)^−^	1.30	11.44
**4**-(Sc_4_F_13_)^−^	1.43	11.73
**5**-(Sc_4_F_13_)^−^	1.64	11.56
**6**-(Sc_4_F_13_)^−^	1.66	11.57
**7**-(Sc_4_F_13_)^−^	1.68	11.93
**8**-(Sc_4_F_13_)^−^	1.71	11.92
**9**-(Sc_4_F_13_)^−^	1.85	11.51
**10**-(Sc_4_F_13_)^−^	2.03	12.28
**11**-(Sc_4_F_13_)^−^	2.38	12.02
**12**-(Sc_4_F_13_)^−^	2.49	12.07
**13**-(Sc_4_F_13_)^−^	4.05	12.38

a The VDEs were calculated at the OVGF/SDD/aug-cc-pVDZ theory level, and Δ*E*s were determined at the CCSD/SDD/aug-cc-pVDZ theory level (both for the geometries optimized with the ωB97XD method and SDD/aug-cc-pVDZ basis sets).

Despite the large vertical excess electron binding energies of the (Sc_*n*_F_3*n*+1_)^−^ anions, the adiabatic electron affinities (AEAs) of their corresponding neutral Sc_*n*_F_3*n*+1_ parents are substantially smaller. Namely, we predicted an AEA of 7.46, 7.57, 8.18, and 9.37 eV for ScF_4_, Sc_2_F_7_, Sc_3_F_10_, and Sc_4_F_13_, respectively. Clearly, the structure reorganization caused by the absence of an excess electron leads to the neutral isomers whose geometrical parameters are significantly different than those of their corresponding daughter anions.

**Figure 5:**
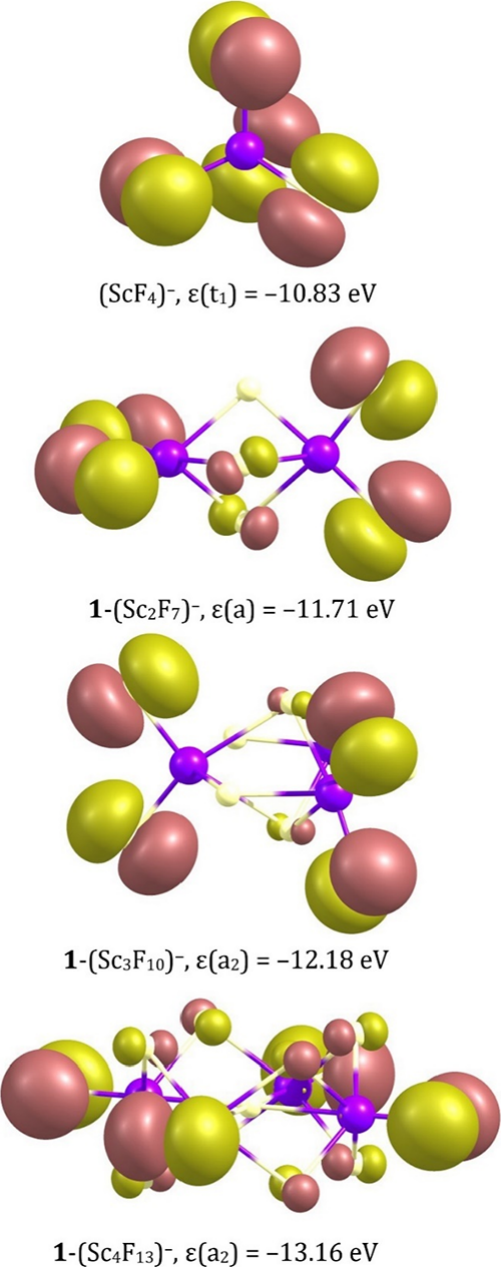
Highest energy canonical molecular orbitals and their corresponding eigenvalues for the most stable isomers of (Sc_*n*_F_3*n*+1_)^−^ anions (*n* = 1–4).

The highest doubly occupied molecular orbitals (HOMO) of the most stable **1**-(Sc_2_F_7_)^−^, **1**-(Sc_3_F_10_)^−^, and **1**-(Sc_4_F_13_)^−^ isomeric structure reveal both (i) the effective delocalization of an excess negative charge among electronegative fluorine ligands and (ii) the absence of destabilizing antibonding ligand-central atom interactions (see Figure 5 where the HOMO for the (ScF_4_)^−^ anion is also shown for comparison).

### Stability of (Sc_*n*_F_3*n*+1_)^−^ Anions (*n* = 2–4) against Fragmentation

In order to verify whether the (Sc_*n*_F_3*n*+1_)^−^ anions are thermodynamically stable, we evaluated the Gibbs free reaction energies at *T* = 298.15 K (Δ*G*_r_^298^) for the most probable fragmentation processes these species might be susceptible to. Namely, we considered two fragmentation channels for each anion: (i) the detachment of the ScF_3_ system (i.e., (Sc_*n*_F_3*n*+1_)^−^ → (Sc_*n*–1_F_3(_*_n__–_*_1)+1_)^−^ + ScF_3_) and (ii) the detachment of the F^–^ anion (i.e., (Sc_*n*_F_3*n*+1_)^−^ → Sc_*n*_F_3*n*_ + F^–^). The results collected in Table 2 indicate that all (Sc_*n*_F_3*n*+1_)^−^ anions considered are thermodynamically stable as the Δ*G*_r_^298^ values corresponding to their fragmentation processes are positive and span the 1.95–5.03 eV range. In particular, the Gibbs free reaction energies predicted for the detachment of the ScF_3_ system from a given (Sc_*n*_F_3*n*+1_)^−^ anion are ca. two times smaller than those predicted for the F^–^ loss, which is likely caused by large electronic stabilities of the (Sc*_n__–_*_1_F_3(_*n*_–1)+1_)^−^ superhalogen anions produced in the former processes. In addition, we verified that the (ScF_4_)^−^ anion is also thermodynamically stable as the Gibbs free reaction energy calculated for the (ScF_4_)^−^ → ScF_3_ + F^–^ process is equal to 4.26 eV.

**Table 2:** Reaction Energies (Δ*E*_r_ in eV) and Gibbs Free Reaction Energies (Δ*G*_r_^298^ in eV) Predicted for the Fragmentation Processes of the (Sc_*n*_F_3*n*+1_)^−^ Anions (*n* = 2–4) at *T* = 298.15 K

fragmentation path	Δ*E*_r_	Δ*G*_r_^298^
(Sc_2_F_7_)^−^ → (ScF_4_)^−^ + ScF_3_	2.34	1.95
(Sc_2_F_7_)^−^ → Sc_2_F_4_ + F^–^	4.57	4.29
(Sc_3_F_10_)^−^ → (Sc_2_F_7_)^−^ + ScF_3_	3.03	2.25
(Sc_3_F_10_)^−^ → Sc_3_F_9_ + F^–^	5.60	5.03
(Sc_4_F_13_)^−^ → (Sc_3_F_10_)^−^ + ScF_3_	3.41	2.59
(Sc_4_F_13_)^−^ → Sc_4_F_12_ + F^–^	4.70	4.26

Hence, we are confident that (Sc_2_F_7_)^−^, (Sc_3_F_10_)^−^, and (Sc_4_F_13_)^−^ anions are stable against fragmentation processes at room temperature.

## Conclusions

On the basis of the ab initio and density functional theory electronic structure calculations carried out using the ωB97XD, CCSD, and OVGF methods with the SDD effective core potentials and the aug-cc-pVDZ basis sets performed for the (Sc_*n*_F_3*n*+1_)^−^ (*n* = 2–4) anions and their corresponding neutral parents, we arrive at the following conclusions:

(Sc_2_F_7_)^−^, (Sc_3_F_10_)^−^, and (Sc_4_F_13_)^−^ anions are thermodynamically stable systems, not susceptible to fragmentations yielding either ScF_3_ or F^–^ products;
The adiabatic electron affinities characterizing neutral Sc_2_F_7_, Sc_3_F_10_, and Sc_4_F_13_ molecules are relatively large (7.57–9.37 eV);
The vertical electron detachment energies of the (Sc_*n*_F_3*n*+1_)^−^ (*n* = 1–4) anions are very large (10.01–12.29 eV) and increase when *n* develops from 1 to 4 (the largest VDE of 12.29 eV corresponds to the most stable isomer of (Sc_4_F_13_)^−^ anion);
The analysis of the isomeric structures of the (Sc_*n*_F_3*n*+1_)^−^ (*n* = 2–4) anions reveals that adopting compact rather than chain-like structures is favored as the former enable the formation of larger number of Sc–F–Sc bridging linkages.

## Supporting Information Available

Cartesian coordinates of all systems (PDF)
