# Supplementary material for: Strongly Bound Polynuclear Anions Comprising Scandium Fluoride Building Blocks
Source: Inorg Chem. 2023 Oct 2;62(41):17022–9. doi: 10.1021/acs.inorgchem.3c02937 (PMC10583212; doi:10.1021/acs.inorgchem.3c02937)
Supplement: Supplementary file 1 — ic3c02937_si_001.pdf [file ic3c02937_si_001.pdf]

Supporting Information

Strongly bound polynuclear anions comprising scandium fluoride building blocks

Iwona Anusiewicz[1], Piotr Skurski[1,2,3,*]

[1] Laboratory of Quantum Chemistry, Faculty of Chemistry, University of Gdańsk, Wita Stwosza 63, 80-308 Gdańsk, Poland

[2] Department of Chemistry, University of Utah, Salt Lake City, Utah 84112, United States

[3] QSAR Lab Ltd., Trzy Lipy 3, 80-172, Poland

[*] corresponding author: piotr.skurski@ug.edu.pl

Table S1. Cartesian coordinates in Å of the isomeric structures of studied anions.

**1**-$(Sc_2F_7)^-$			
21	0.000000000	1.490508000	0.006591000
21	0.000000000	-1.490508000	0.006591000
9	-1.260338000	-0.186471000	-0.792929000
9	1.142905000	-2.683744000	-0.910500000
9	1.260338000	0.186471000	-0.792929000
9	-1.142905000	2.683744000	-0.910500000
9	1.077108000	2.704064000	0.962846000
9	-1.077108000	-2.704064000	0.962846000
9	0.000000000	0.000000000	1.450407000
**2**-$(Sc_2F_7)^-$			
21	0.296394000	-1.521642000	0.000000000
21	-0.321705000	1.737654000	0.000000000
9	1.181687000	0.462480000	0.000000000
9	2.039628000	-2.254740000	0.000000000
9	-0.454588000	2.777580000	1.555956000
9	-0.454588000	-2.181140000	1.602853000
9	-0.454588000	2.777580000	-1.555956000
9	-0.454588000	-2.181140000	-1.602853000
9	-1.343903000	0.095351000	0.000000000
**3**-$(Sc_2F_7)^-$			
21	0.000000000	2.029511000	-0.000053000
21	0.000000000	-2.029511000	-0.000053000
9	-0.898722000	2.587784000	1.554651000
9	-1.795196000	-2.589847000	0.000418000
9	0.000000000	0.000000000	-0.000589000
9	-0.898086000	2.589149000	-1.554650000
9	0.898722000	-2.587784000	1.554651000
9	0.898086000	-2.589149000	-1.554650000
9	1.795196000	2.589847000	0.000418000
**1**-$(Sc_3F_{10})^-$			
21	0.000000000	1.661986000	-0.801311000
21	0.000000000	-1.661986000	-0.801311000
21	0.000000000	0.000000000	1.827119000
9	0.000000000	-1.944407000	1.300221000
9	0.000000000	0.000000000	3.700043000
9	0.000000000	0.000000000	-2.004597000
9	1.488649000	-2.655443000	-1.337023000
9	-1.488649000	-2.655443000	-1.337023000
9	-1.488649000	2.655443000	-1.337023000
9	-1.279770000	0.000000000	0.264189000
9	0.000000000	1.944407000	1.300221000
9	1.279770000	0.000000000	0.264189000
9	1.488649000	2.655443000	-1.337023000
**2**-$(Sc_3F_{10})^-$			
21	0.000000000	1.980413000	0.003224000
21	1.715088000	-0.990207000	0.003224000
21	-1.715088000	-0.990207000	0.003224000
9	-2.568263000	-1.482788000	1.585067000
9	-2.785298000	-1.608092000	-1.399112000
9	0.000000000	0.000000000	0.949450000
9	0.000000000	-1.995960000	-0.509962000
9	0.000000000	3.216185000	-1.399112000

9	2.785298000	-1.608092000	-1.399112000
9	0.000000000	2.965575000	1.585067000
9	2.568263000	-1.482788000	1.585067000
9	1.728552000	0.997980000	-0.509962000
9	-1.728552000	0.997980000	-0.509962000
**3**-$(Sc_3F_{10})^-$			
21	0.000000000	0.000000000	0.197491000
21	0.000000000	2.927358000	-0.073578000
21	0.000000000	-2.927358000	-0.073578000
9	-0.746163000	1.412595000	1.371812000
9	-0.822242000	-3.926700000	-1.425580000
9	0.822242000	3.926700000	-1.425580000
9	1.448864000	1.467528000	0.160582000
9	0.777584000	-4.270615000	0.982457000
9	0.802691000	-1.375706000	-1.147993000
9	-0.802691000	1.375706000	-1.147993000
9	0.746163000	-1.412595000	1.371812000
9	-1.448864000	-1.467528000	0.160582000
9	-0.777584000	4.270615000	0.982457000
**4**-$(Sc_3F_{10})^-$			
21	-1.659608000	0.125830000	-1.077357000
21	1.659608000	-0.125830000	-1.077357000
21	0.000000000	0.000000000	2.236695000
9	-0.348346000	1.487196000	3.295536000
9	0.000000000	1.242651000	-1.241189000
9	1.453358000	0.392610000	0.944281000
9	0.348346000	-1.487196000	3.295536000
9	2.864289000	-1.500732000	-0.646363000
9	-2.566557000	-0.778038000	-2.447910000
9	-1.453358000	-0.392610000	0.944281000
9	-2.864289000	1.500732000	-0.646363000
9	2.566557000	0.778038000	-2.447910000
9	0.000000000	-1.242651000	-1.241189000
**5**-$(Sc_3F_{10})^-$			
21	1.927685000	-2.106169000	0.000000000
21	-2.627583000	0.711979000	0.000000000
21	0.550896000	1.476602000	0.000000000
9	1.069598000	2.332331000	1.570434000
9	-1.433837000	2.288321000	0.000000000
9	3.748639000	-2.580793000	0.000000000
9	1.069598000	2.332331000	-1.570434000
9	1.069598000	-2.703076000	1.564116000
9	-3.632900000	0.532759000	-1.555171000
9	-3.632900000	0.532759000	1.555171000
9	-0.836981000	-0.150512000	0.000000000
9	1.069598000	-2.703076000	-1.564116000
9	1.857260000	-0.073338000	0.000000000
**6**-$(Sc_3F_{10})^-$			
21	0.000000000	3.481395000	-0.662251000
21	0.000000000	0.000000000	1.351773000
21	0.000000000	-3.481395000	-0.662251000
9	-1.540906000	0.000000000	2.376285000
9	-1.562856000	3.608506000	-1.689522000
9	0.000000000	4.641016000	0.811682000
9	1.540906000	0.000000000	2.376285000

9	1.562856000	3.608506000	-1.689522000
9	0.000000000	-4.641016000	0.811682000
9	-1.562856000	-3.608506000	-1.689522000
9	1.562856000	-3.608506000	-1.689522000
9	0.000000000	1.591825000	0.159260000
9	0.000000000	-1.591825000	0.159260000
**1**-$(Sc_4F_{13})^-$			
21	0.000000000	2.177989000	-0.000010000
21	0.000000000	-2.177989000	-0.000010000
21	-2.177948000	0.000000000	-0.000107000
21	2.177948000	0.000000000	-0.000107000
9	1.534323000	1.534356000	-1.240441000
9	1.534285000	1.534283000	1.240313000
9	0.000000000	0.000000000	-0.000155000
9	-4.038232000	0.000000000	0.000170000
9	-1.534323000	-1.534356000	-1.240441000
9	1.534323000	-1.534356000	-1.240441000
9	-1.534285000	-1.534283000	1.240313000
9	0.000000000	4.038272000	0.000437000
9	4.038232000	0.000000000	0.000170000
9	-1.534323000	1.534356000	-1.240441000
9	1.534285000	-1.534283000	1.240313000
9	-1.534285000	1.534283000	1.240313000
9	0.000000000	-4.038272000	0.000437000
**2**-$(Sc_4F_{13})^-$			
21	-1.614492000	0.000000000	-0.835945000
21	1.614492000	0.000000000	-0.835945000
21	0.000000000	2.286548000	0.788592000
21	0.000000000	-2.286548000	0.788592000
9	0.000000000	-3.997631000	0.054018000
9	-1.929619000	-1.622421000	0.302008000
9	0.000000000	-2.504294000	2.633148000
9	-2.884249000	0.000000000	-2.182564000
9	1.929619000	1.622421000	0.302008000
9	0.000000000	-1.248053000	-1.391706000
9	0.000000000	3.997631000	0.054018000
9	0.000000000	0.000000000	0.787159000
9	0.000000000	1.248053000	-1.391706000
9	-1.929619000	1.622421000	0.302008000
9	1.929619000	-1.622421000	0.302008000
9	0.000000000	2.504294000	2.633148000
9	2.884249000	0.000000000	-2.182564000
**3**-$(Sc_4F_{13})^-$			
21	2.055176000	0.890536000	0.472959000
21	-0.642790000	2.106456000	-0.451095000
21	-2.269226000	-0.692664000	0.398795000
21	0.936343000	-2.032469000	-0.395437000
9	-2.270226000	1.409125000	0.467645000
9	-0.584064000	0.092834000	-0.794412000
9	0.472422000	2.053784000	1.217534000
9	-3.620869000	-1.016415000	-0.836608000
9	-2.892964000	-0.914674000	2.142233000
9	1.901172000	-3.445130000	0.343053000
9	1.083950000	-0.629813000	1.285425000
9	3.624054000	1.505056000	1.249705000

9	2.410967000	-0.582558000	-0.817367000
9	1.303065000	1.973693000	-1.093349000
9	-1.237928000	3.622468000	-1.343094000
9	-0.979434000	-2.245005000	0.305823000
9	0.604346000	-2.457702000	-2.185439000
**4**-$(Sc_4F_{13})^-$			
21	-1.499302000	0.975315000	0.466413000
21	1.499302000	-0.975315000	0.466413000
21	2.042300000	2.087775000	-0.433288000
21	-2.042300000	-2.087775000	-0.433288000
9	0.000000000	2.147721000	-0.255668000
9	-2.688916000	2.288543000	1.025744000
9	-2.934444000	-2.504097000	-2.020065000
9	-2.587691000	-0.684192000	0.977047000
9	0.000000000	0.000000000	1.464554000
9	2.587691000	0.684192000	0.977047000
9	2.393639000	3.564776000	0.657308000
9	-2.393639000	-3.564776000	0.657308000
9	-1.592511000	-0.068380000	-1.193936000
9	0.000000000	-2.147721000	-0.255668000
9	2.688916000	-2.288543000	1.025744000
9	2.934444000	2.504097000	-2.020065000
9	1.592511000	0.068380000	-1.193936000
**5**-$(Sc_4F_{13})^-$			
21	-0.769224000	-0.513834000	-0.759169000
21	1.403626000	1.706042000	-0.211009000
21	2.945248000	-0.946652000	0.490078000
21	-3.682742000	-0.108894000	0.494895000
9	0.953754000	-1.248879000	0.087196000
9	-1.126728000	-1.837254000	-2.012133000
9	-5.173305000	-1.127920000	0.046667000
9	-2.614753000	0.488733000	-1.066801000
9	0.442332000	0.776991000	-1.778305000
9	3.013765000	0.645289000	-0.862501000
9	3.760189000	-2.146046000	-0.688812000
9	-4.094708000	1.181498000	1.769303000
9	-2.032584000	-1.182442000	0.789401000
9	-0.396226000	1.167506000	0.513467000
9	1.630922000	3.546284000	-0.372593000
9	3.706497000	-1.443807000	2.125833000
9	2.171392000	0.861168000	1.414754000
**6**-$(Sc_4F_{13})^-$			
21	-1.816475000	-0.882994000	0.000000000
21	0.146605000	0.908489000	2.319102000
21	1.710201000	-1.036283000	0.000000000
21	0.146605000	0.908489000	-2.319102000
9	0.128456000	0.521660000	0.000000000
9	-1.252868000	-0.487698000	-1.958534000
9	-1.252868000	-0.487698000	1.958534000
9	0.351520000	0.720596000	4.158427000
9	0.048877000	2.735675000	-2.016884000
9	0.048877000	2.735675000	2.016884000
9	3.175628000	-2.171291000	0.000000000
9	1.866465000	-0.103224000	-1.758931000
9	0.072781000	-2.074187000	0.000000000

9	0.351520000	0.720596000	-4.158427000
9	-2.928613000	0.623421000	0.000000000
9	1.866465000	-0.103224000	1.758931000
9	-2.912423000	-2.391605000	0.000000000
**7**-$(Sc_4F_{13})^-$			
21	1.254776000	0.614131000	0.000000000
21	-0.328020000	-1.881877000	1.707774000
21	-0.328020000	-1.881877000	-1.707774000
21	-0.589350000	3.251169000	0.000000000
9	-0.616654000	-0.468934000	0.000000000
9	1.358726000	-0.727203000	-1.532203000
9	-2.443857000	3.300678000	0.000000000
9	0.304650000	2.002275000	-1.268863000
9	2.971739000	1.317567000	0.000000000
9	1.358726000	-0.727203000	1.532203000
9	0.394194000	-3.234011000	-2.771929000
9	0.153676000	4.954515000	0.000000000
9	0.304650000	2.002275000	1.268863000
9	-1.799737000	-1.224089000	2.638904000
9	0.394194000	-3.234011000	2.771929000
9	-1.799737000	-1.224089000	-2.638904000
9	-0.602471000	-2.974711000	0.000000000
**8**-$(Sc_4F_{13})^-$			
21	2.733189000	0.043293000	0.487418000
21	-2.586352000	-0.484010000	0.541181000
21	0.324240000	-1.841761000	-0.580999000
21	-0.475925000	2.143518000	-0.467961000
9	1.524122000	-1.610989000	1.000624000
9	1.346198000	1.502368000	0.156942000
9	-0.780833000	-0.106409000	-0.750695000
9	-0.677435000	2.367826000	-2.301396000
9	-1.344351000	-2.119900000	0.565780000
9	-2.104781000	1.496711000	0.593754000
9	0.328956000	-3.381288000	-1.611549000
9	-0.404090000	3.858843000	0.246865000
9	-3.323613000	-0.659364000	2.241245000
9	-3.867642000	-0.779159000	-0.769845000
9	3.077188000	0.431404000	2.280488000
9	4.419814000	0.076293000	-0.304740000
9	1.817783000	-0.752096000	-1.299964000
**9**-$(Sc_4F_{13})^-$			
21	0.000000000	2.797689000	-0.249939000
21	0.000000000	-2.797689000	-0.249939000
21	0.000000000	0.000000000	-1.468941000
21	0.000000000	0.000000000	1.822681000
9	0.697602000	1.903002000	1.547109000
9	1.379720000	1.371672000	-1.061222000
9	-1.043808000	0.768144000	0.256038000
9	1.043808000	-0.768144000	0.256038000
9	0.813360000	-1.720423000	-2.070437000
9	-1.379720000	-1.371672000	-1.061222000
9	-1.586630000	3.595110000	0.343070000
9	1.067372000	4.263214000	-0.683707000
9	-0.813360000	1.720423000	-2.070437000
9	-0.697602000	-1.903002000	1.547109000

9	1.586630000	-3.595110000	0.343070000
9	-1.067372000	-4.263214000	-0.683707000
9	0.000000000	0.000000000	3.679286000
**10**-$(Sc_4F_{13})^-$			
21	0.372568000	-0.119540000	2.071944000
21	0.372568000	-0.119540000	-2.071944000
21	-0.382895000	-2.805229000	0.000000000
21	-0.365212000	3.090769000	0.000000000
9	-0.696437000	-0.045571000	-3.593472000
9	-0.696437000	-0.045571000	3.593472000
9	-2.138694000	3.638423000	0.000000000
9	-2.236597000	-2.669525000	0.000000000
9	-0.107236000	-0.439734000	0.000000000
9	-0.018015000	1.838068000	1.492295000
9	0.279783000	-2.156586000	-1.790054000
9	0.279783000	-2.156586000	1.790054000
9	-0.018015000	1.838068000	-1.492295000
9	0.816405000	4.523562000	0.000000000
9	0.157392000	-4.586870000	0.000000000
9	2.192499000	0.076959000	2.383841000
9	2.192499000	0.076959000	-2.383841000
**11**-$(Sc_4F_{13})^-$			
21	-0.282436000	-2.293831000	1.737590000
21	0.968092000	0.338603000	0.000000000
21	-0.282436000	-2.293831000	-1.737590000
21	-0.360660000	4.139715000	0.000000000
9	0.049553000	-0.246685000	1.700211000
9	0.049553000	-0.246685000	-1.700211000
9	2.767868000	0.714329000	0.000000000
9	0.134137000	2.143343000	0.000000000
9	-2.233087000	4.233474000	0.000000000
9	0.401764000	4.843277000	1.562855000
9	1.000435000	-3.366614000	2.540178000
9	-1.783940000	-2.297800000	2.829432000
9	-1.783940000	-2.297800000	-2.829432000
9	1.000435000	-3.366614000	-2.540178000
9	1.037790000	-1.748589000	0.000000000
9	0.401764000	4.843277000	-1.562855000
9	-1.141641000	-2.951779000	0.000000000
**12**-$(Sc_4F_{13})^-$			
21	4.380116000	0.010012000	-0.002897000
21	0.326491000	0.043664000	0.007643000
21	-2.237423000	-1.705738000	-0.001430000
21	-2.316647000	1.665318000	-0.000970000
9	-0.098461000	-1.895545000	0.003837000
9	4.960711000	1.734824000	0.436001000
9	-1.171106000	0.007724000	-1.278998000
9	-2.726201000	-2.695251000	-1.495625000
9	-1.175699000	0.008781000	1.286205000
9	-0.188636000	1.960703000	0.002854000
9	-3.439549000	-0.047218000	-0.002004000
9	4.788961000	-0.496474000	-1.759593000
9	-2.856011000	2.632134000	1.491162000
9	4.824784000	-1.271909000	1.287633000
9	-2.851135000	2.631092000	-1.495456000

9	-2.732078000	-2.694645000	1.491333000
9	2.308500000	0.094851000	0.027175000
**13**-$(Sc_4F_{13})^-$			
21	-1.218293000	-1.609460000	0.003045000
21	1.218293000	1.609460000	0.003045000
21	-0.859681000	5.051946000	-0.003126000
21	0.859681000	-5.051946000	-0.003126000
9	2.230818000	1.553968000	-1.538358000
9	-2.230818000	-1.553968000	-1.538358000
9	0.000000000	-3.170730000	-0.002154000
9	2.223194000	1.558257000	1.549600000
9	-2.223194000	-1.558257000	1.549600000
9	0.610443000	6.213975000	-0.002176000
9	-1.881487000	5.136806000	-1.570534000
9	-1.883821000	5.136846000	1.562782000
9	0.000000000	0.000000000	0.002053000
9	0.000000000	3.170730000	-0.002154000
9	-0.610443000	-6.213975000	-0.002176000
9	1.881487000	-5.136806000	-1.570534000
9	1.883821000	-5.136846000	1.562782000
